# Antibiotic prescribing for acute, non-complicated infections in primary care in Germany: baseline assessment in the cluster randomized trial ARena

**DOI:** 10.1186/s12879-021-06571-0

**Published:** 2021-08-26

**Authors:** Regina Poss-Doering, Dorothea Kronsteiner, Martina Kamradt, Edith Andres, Petra Kaufmann-Kolle, Michel Wensing, ARena-study group, Joachim Szecsenyi, Michel Wensing, Martina Kamradt, Regina Poß-Doering, Dorothea Kronsteiner, Petra Kaufmann-Kolle, Edith Andres, Veit Wambach, Joerg Lindenthal, Julian Bleek, Alexander Günter, Lutz Bader, Joachim Szecsenyi

**Affiliations:** 1 Department of General Practice and Health Services Research, University Hospital Heidelberg, Im Neuenheimer Feld 130.3, 69120 Heidelberg, Germany; 2 IMBI Institute for Medical Biometry, University Hospital Heidelberg, Heidelberg, Germany; 3 aQua Institut, Goettingen, Germany

**Keywords:** Antimicrobial resistance, Antibiotic prescribing, Acute upper respiratory tract infections, Primary care, Mixed logistic regression model

## Abstract

**Background:**

Antimicrobial resistance is fueled by inappropriate use of antibiotics. Global and national strategies support rational use of antibiotics to retain treatment options and reduce resistance. In Germany, the ARena project (Sustainable reduction of antibiotic-induced antimicrobial resistance) intended to promote rational use of antibiotics for acute non-complicated infections by addressing network-affiliated physicians, primary care teams and patients through multiple interacting interventions. The present study documented patterns of antibiotic prescribing for patients with acute non-complicated infections who consulted a physician in these networks at the start of the ARena project. It explored variation across subgroups of patients and draws comparisons to prescribing patterns of non-targeted physicians.

**Methods:**

This retrospective cross-sectional analysis used mixed logistic regression models to explore factors associated with the primary outcome, which was the percentage of patient cases with acute non-complicated respiratory tract infections consulting primary care practices who were treated with antibiotics. Secondary outcomes concerned the prescribing of different types of antibiotics. Descriptive methods were used to summarize the data referring to targeted physicians in primary care networks, non-targeted physicians (reference group), and patient subgroups.

**Results:**

Overall, antibiotic prescribing rates were 32.0% in primary care networks and 31.7% in the reference group. General practitioners prescribed antibiotics more frequently than other medical specialist groups (otolaryngologists vs. General practitioners OR = 0.465 CI = [0.302; 0.719], p < 0.001, pediatricians vs. General practitioners: OR = 0.369 CI = [0.135; 1.011], p = 0.053). Quinolone prescribing rates were 9.9% in primary care networks and 8.1% in reference group. Patients with comorbidities had a higher likelihood of receiving an antibiotic and quinolone prescription and were less likely to receive a guideline-recommended substance. Younger patients were less likely to receive antibiotics (OR = 0.771 CI = [0.636; 0.933], p = 0.008). Female gender was more likely to receive an antibiotic prescription (OR = 1.293 CI = [1.201, 1.392], p < 0.001).

**Conclusion:**

This study provided an overview of observed antibiotic prescribing for acute non-complicated respiratory tract infections in German primary care at the start of the ARena project. Findings indicate potential for improvement and will serve as comparator for the post-interventional outcome evaluation to facilitate describing of potential changes.

**Supplementary Information:**

The online version contains supplementary material available at 10.1186/s12879-021-06571-0.

## Background

The use of antibiotics in German primary care is lower than average in other countries [1, 2] but there is still potential for lowering prescribing rates for specific conditions. In light of growing antimicrobial resistance, the holistic German national antibiotic resistance strategy (DART 2020) follows the One Health approach to promote awareness, counteract microbial resistance and preserve antibiotic treatment options [3]. Monitoring the use of antibiotics is an important strategy to reduce the spread of antimicrobial resistance, particularly in primary care where about 85% of the used antibiotics in Germany are prescribed [4, 5]. Antibiotics are prescribed in 41% of GP consultations for acute respiratory tract infections (ARTI) [4]. Only 52% of these prescriptions are in accordance with prevailing clinical recommendations [6].

In recent years, several research projects tested strategies that aim to enhance the rational and appropriate use of antibiotics in healthcare. In this context, the three-armed, cluster randomized trial ARena (Sustainable reduction of antibiotic-induced antimicrobial resistance; conducted from 2017 to 2020) aimed to foster the rational use of antibiotics for acute non-complicated respiratory tract infections in primary care in Germany [7]. By applying a multifaceted strategy with multiple interacting intervention components, ARena addressed primary care physicians, care teams as well as patients [7]. An innovative aspect of ARena was its embedding in 14 primary care networks (PCNs) across two German federal states. PCNs can be described as formalized collaborations of economically independent physicians and other healthcare providers working in single or joint practices who interact regularly, share patients, standardize treatment and care according to evidence-based practice guidance, regularly attend continuing education, and discuss concerns if these arise. They give support regarding practice management and quality improvement [8] and therefore were expected to amplify the impact of the ARena implementation program. More detailed description of the study design and interventions can be found elsewhere [7].

Previous research investigated the development of antibiotic prescribing rates in primary care and found a relatively stable utilization in Germany from 2008 to 2014 [9]. However, strong variations of overall and age-group-specific distributions of antibiotic subgroups could be identified [10]. A strong awareness of antimicrobial resistance has been observed among German General Practitioners (GPs), while measures to improve rational prescribing were found to be not widely implemented [11]. In more recent research, a 5-year cohort study of antibiotic prescribing rates by family physicians in Ontario, Canada, aimed to describe predictors of antibiotic prescribing and inter-physician variability in antibiotic prescribing. It was concluded that observed substantial inter-physician variability in antibiotic prescribing could not be explained by sociodemographic and clinical patient characteristics [12]. Further recent research also found that the use of antibiotics in German primary care showed large variations between and within medical specialties and seasons, and that a considerable proportion of antibiotic prescribing lacked conformity with national guideline recommendations [13]. Though these study findings might not reflect the most current situation or guideline-conformity of prescribing, there is an indication that antibiotics are still not prescribed appropriately regarding indication and spectrum and thus are frequently used inappropriately. For targeted quality improvement, it is relevant to know which subgroups of patients and physicians are at highest risk of inappropriate utilization of antibiotics.

For the ARena study, the outcome evaluation is based on quarterly claims-data as provided by a large German statutory health insurer and references established indicators of the European Surveillance of Antimicrobial Consumption Network (ESAC-Net) [14] which were tailored to the specifics of the study. Primary and secondary outcomes are related to general prescribing of systemic antibiotics as well as indication-specific prescribing of antibiotics currently recommended [15, 16] by German clinical guidelines [15, 16]. To explore the actual antibiotic prescribing rates in the participating PCNs and their determinants, this present study aimed to define the baseline antibiotic prescribing rates as percentage of cases with acute non-complicated respiratory tract infections prior to the start of the ARena project and explore potential percentage variation across the intervention arms, medical specialties and subgroups of patients. Also, this baseline situation was to be compared to a non-PCN standard care reference group (RG), using the provided claims data and considering characteristics such as patient age, gender and insurance and health status.

## Methods

### Study design

This retrospective cross-sectoral analysis was based on claims data regarding antibiotic prescribing in primary care at the start of a prospective interventional trial (Q3 2016–Q2 2017). The outcomes evaluation (Trial registration: ISRCTN, ISRCTN58150046) is designed as a three-armed cluster randomized trial with fourteen PCNs and RG that reflects standard care in two German federal states (Bavaria and North Rhine-Westphalia). The ARena-study was approved by the ethics committee of the Medical Faculty of the University of Heidelberg (reference number: S-353/2017). The study was planned with an intervention period (Q4 2017–Q2 2019), and two parts of evaluation: (a) an outcome evaluation based on quarterly claims-data (two measuring points: baseline Q3 2016–Q2 2017; post-interventional Q3 2018–Q2 2019), and (b) a process evaluation based on surveys [7].

### Study population

All PCNs situated in Bavaria and North Rhine-Westphalia with a valid contract with AOK referring to a specific healthcare delivery program (defined by German law § 140a SGB V a.F. and § 140a Abs. 1. S. 2 Alt. 1 SGB V n.F) were contacted by mail by the aQua Institute, Göttingen, Germany, for study participation. Fourteen PCNs were recruited to participate in the ARena study and randomized to the intervention arms (arm I = 4 PCNs; arm II = 5 PCNs; arm III = 5 PCNs). For administrative reasons, the focus was on their patients insured by the statutory health insurer AOK and registered within the specific healthcare delivery program. Around 35% of the population within geographic reach of the participating PCNs in Bavaria alone are insured by AOK [7]. At baseline, approximately 40,000 patients with AOK health insurance were registered in 196 participating primary care practices in these 14 networks. Medical specialties of the participating physicians included (n = 309) were general practice, otolaryngology, pediatrics, urology and gynecology. The patient population for the three intervention arms comprised patient cases who sought primary care for one of the following reasons: acute non-complicated upper respiratory tract infections (URTI), bronchitis, sinusitis, tonsillitis and otitis media. Thus, patient study populations differed per indexed consultation reason regarding number of cases, age of patients and insurance status. The diagnoses were based on physician-recorded ICD-10 codes and the prescribing information in administrative data provided by the statutory health insurer AOK for quarterly reimbursement periods which were linked by the pseudonymized patient individual insurance number. Each physician-recorded ICD-10 code for the defined index diagnoses represents a case, where each patient can produce multiple cases. A patients’ case is recorded for each ICD-10 code within each quarter. Prescribing information was derived from quarterly claims data as provided by the health insurer and included patient cases were not actively recruited. Consent for data inclusion in the analysis was obtained, an additional written informed patient consent was prerequisite in North Rhine-Westphalia.

### Measures

The primary outcome in this study was the baseline prescribing rate as percentage of cases of all patients with acute respiratory tract infections consulting primary care practices who were treated with systemic antibiotics without pathogen detection. More precise, patients suffering from acute bronchitis (18–75 years), sinusitis (> 18 years), otitis media (> 2 years), acute URTI (acute rhino-pharyngitis, pharyngitis) (> 1 year), or tonsillitis (> 1 year) were considered in the primary outcome. Due to the structure of the provided quarterly claims data and data protection regulations, ICD-10 codes and antibiotic prescriptions were matched for each quarter of year, which means that each patient can provide one case per quarter because the direct connection of ICD-10 code and antibiotic prescription is not possible. Physician-recorded diagnoses that warrant antibiotic therapy were not included for analysis. Specifically excluded were diagnoses for streptococcal tonsilitis and other pathogen-caused acute forms of tonsillitis. (Additional file 1: Table S1 details included diagnoses and related ICD-10 codes, Table S2 provides a list of excluded diagnoses.)

The following secondary outcomes regarding acute non-complicated infections were examined:

1. Percentage of cases who received guideline-recommended antibiotics for acute
  URTI (> 1 year; recommendation: amoxicillin)
  Bronchitis (18–75 years; recommendation: amoxicillin, tetracycline, macrolides)
  Sinusitis (> 18 years; recommendation: amoxicillin, cefuroxime)
  Tonsillitis (> 1 year; recommendation: penicillin, erythromycin)
  Otitis- Otitis media (> 2 years; recommendation: amoxicillin, erythromycin, cefuroxime)
2. Percentage of cases who received a prescription for quinolones
3. Consumption of broad-spectrum antibiotics in Defined daily dose (DDD%) on practice level (beta-lactam-beta-lactamase inhibitor combinations, cephalosporines of 2nd, 3rd and 4th generation, macrolides (excluding Erythromycin) and gyrase inhibitors (fluoroquinolones)

Further secondary outcomes considered by the study protocol referred to cystitis and community-aquired pneumonia and were not explored due to very small numbers of recorded cases. Patient age specifications for all observed sub-groups follow the study protocol [7]. Categorization of recommended antibiotics was based on existing evidence-based clinical guidelines developed by the German College of General Practitioners and Family Physicians (DEGAM) [16] and the Association of the Scientific Medical Societies in Germany (AWMF) [15]. (See Additional file 1: Table S3 for currently recommended and alternative antibiotics.)

Regarding patients, the following sociodemographic, disease, and treatment characteristics in the claims data provided by AOK were included: age, sex, Charlson comorbidity index (CCI) (predicts 1-year survival in patients based on sum of relevant comorbidities [17, 18], employment status, nationality (missing values aggregated to ‘other’), insurance status (main member, family, retiree), participation in a disease management program (DMP) which is a structured treatment plan to support management of chronic disease and maintain and improve quality of life [19] (type 1 diabetes, type 2 diabetes, coronary heart disease, breast cancer, bronchial asthma, COPD, cardiac insufficiency), classified degree of necessary nursing care [20], and season. Regarding primary care practices, type of location (urban, increasingly urbanized, countryside), type of practice (single or group) and medical specialty group are documented.

### Data analysis

The baseline data were analyzed regarding the four quarters prior to the ARena intervention (Q3 2016–Q2 2017) with a focus on prescribing rates of antibiotics for acute non-complicated self-limiting infections in the intervention arms and the RG that reflects standard care. In addition, patients’ sociodemographic and disease-specific characteristics in either of these groups are summarized. Also focused are the use of guideline-recommended indication-appropriate antibiotics and the group of quinolones.

The primary and all secondary outcomes, as well as all documented data (patient characteristics, disease characteristics, treatment data, and practice characteristics), were first analyzed descriptively. For continuous variables, mean and standard deviation, median, 25%/75%-quantiles [Q1–Q3], min and max are provided, for categorical variables absolute and relative frequencies are given. Note that the description of patient and disease characteristics, as well as treatment data and practice characteristics differ between outcomes, because the considered cases are defined for each outcome by respective index diagnoses and antibiotic prescribing. Therefore, the descriptive analysis is done for each outcome individually. For the subgroups gender, DMP and the CCI, the primary and all secondary outcomes are contrasted using descriptive methods based on patients in PCNs and RG.

A logistic mixed effects regression model was used to investigate factors which may influence the primary outcome. The model considers the nested structure of the data with patients nested in practices, which means practice is included as random effect in the logistic mixed effects model. As fixed effects, medical specialty group (German: Fachgruppe—FGR), urbanization, age group, sex, and the CCI as indication of health status are considered, the selection is based on clinical expertise. Secondary outcomes are analyzed using mixed logistic (1.–2.) or beta regression models (3.). Adjustment is done as described for the primary outcome model. Since this is an explorative study, all p-values do not have confirmatory value.

## Results

### Sociodemographic characteristics

The primary analysis considered a total of 3,129,289 cases in PCNs (n = 18,207) and the RG (n = 3,111,082) diagnosed with one of the indexed infections (tonsillitis, sinusitis, otitis media, bronchitis, and URTI). In PCNs, 92.7% of the participating practices were General Practitioners, 4.9% Otolaryngologists, and 2.1% were Pediatricians. In the RG, 73.3% were General Practitioners, 10.6% Otolaryngologists and 14.5% were Pediatricians. The total number of observed cases mentioned above (n = 3,129,289) reflects 2 102 783 patients with a mean of 1.5 cases per patient. In PCNs, 62.8% of the included cases were seen in rural area practices compared to 32.6% of cases in the RG. Mean age of patients was higher in PCNs. In both groups, sex was equally distributed. Patient nationality was not reported for less than 1% of the cases and all cases without this information are considered in the category “other”. Cases in PCNs were older and with a higher morbidity, except for Otitis media where cases were included from the age of two and above. A main group difference in terms of insurance status was apparent in the subgroup of retired insurance members (RG: 10.3%; PCNs 20.8%). Distribution of cases with indexed diagnoses and sociodemographic characteristics of patients in PCNs and RG are presented in Table 1.

**Table 1:** Sociodemographic characteristics of all cases in RG and PCNs (n = 3,129,289) and distribution per indexed diagnoses

Cases	RG	PCNs
Age: Mean (SD)	34.3 (21.07)	47.5 (18.94)
Sex: Female n (%)	1,633,772 (52.5)	10,594 (58.2)
Nationality: n (%)		
German	2,353,362 (75.6)	15,980 (87.8)
Eastern European	548,307 (17.6)	1630 (9.0)
Southern European	109,871 (3.5)	355 (1.9)
Northern European	29,508 (0.9)	79 (0.4%)
Other	70,034 (2.3)	163 (0.9)
Insurance status: n (%)		
Main member	1,857,770 (59.7)	12,278 (67.4)
Family member	876,894 (28.2)	2024 (11.1)
Retired member	319,600 (10.3)	3787 (20.8)
Employment “yes”: n (%)	1,881,657 (60.5)	12,143 (66.7)
Upper respiratory tract infections %	69.2	65.3
Bronchitis %	24.1	29.3
Sinusitis %	7.7	10.1
Tonsilitis %	8.6	5.6
Otitis media %	7.0	4.7

### Primary outcome

The observed baseline antibiotic prescribing rate as percentage of all cases with acute non-complicated respiratory tract infections was slightly higher in PCNs (32.0%) than in RG (31.7%). Across all observed infections and cases, GPs were the largest group of treating physicians to prescribe antibiotics. In PCNs, the percentage ranged from 87.3 to 99.7%, in the RG the range was lower and between 46.9 and 96.7% (See Additional file 1: Table S5 for details on the overall distribution of medical specialty of antibiotics prescribing physicians.). In mixed logistic regression models for the primary outcome in PCNs, the specialist group otolaryngologists (OR = 0.465 CI = [0.302; 0.719], p-value < 0.001) and pediatricians (OR = 0.369 CI = [0.135; 1.011], p-value = 0.007) appeared to prescribe antibiotics less frequently compared to GPs (see Table 2). Looking at patients, women were more likely to receive antibiotics compared to men (OR = 1.293 CI: [1.201; 1.392], p-value < 0.001). Patients under 18 years were less likely than patients aged 18 to 65 to receive an antibiotic prescription (OR = 0.771 CI: [

**Table 2:** Results of the logistic mixed effects regression model for prescribing of antibiotics in PCNs for acute non-complicated respiratory tract infections

	Odds ratio	Lower CI limit	Upper CI limit	Standard error	p-value
Otolaryngologists vs general practitioner	0.465	0.302	0.719	0.222	< 0.001
Pediatrician vs general practitioner	0.369	0.135	1.011	0.514	0.053
Other specialty groups vs general practitioner	0.251	0.075	0.844	0.618	0.026
Increasingly urbanized vs rural location	0.832	0.512	1.351	0.248	0.457
Urban vs rural location	0.901	0.693	1.192	0.138	0.489
PCN size medium vs small	0.960	0.634	1.453	0.212	0.846
PCN size large vs small	1.009	0.671	1.517	0.208	0.967
Patient age < 18 vs 18–65	0.771	0.636	0.933	0.098	0.008
Patient age > 65 vs 18–65	1.077	0.967	1.200	0.055	0.179
Female patients vs male patients	1.293	1.201	1.392	0.038	< 0.001
Charlson Index 1 and 2 vs 0	1.562	1.436	1.700	0.043	< 0.001
Charlson Index 3 and 4 vs 0	1.662	1.435	1.925	0.075	< 0.001
Charlson Index > = 5 vs 0	1.760	1.505	2.059	0.080	< 0.001

*vs* versus, *PCN* primary care network

0.636; 0.933], p-value = 0.008). An increased CCI implied higher prescribing rates. Table 2 shows results of the mixed logistic regression model for antibiotic prescribing rates in the PCNs for acute upper respiratory tract infections using practice and patient-related characteristics as covariates.

For the DMPs for Type 2 Diabetes mellitus, asthma, COPD and coronary heart disease, the inclusion of cases across all PCNs was > 90%. In the RG, highest inclusion was for the DMP Asthma (76.8% of the observed cases). In PCNs, 46% of observed cases were registered in a Type 1 Diabetes mellitus DMP, in RG only 17.7% of the cases were in a DMP. In PCNs, 10.9% of cases were in a breast cancer DMP, in RG this percentage was 5.1%.

The different levels of needed nursing care (level 1 to 5) were equally distributed across groups. Additionally, the CCI of patients in PCNs showed higher relative frequencies in high index values and lower relative frequencies in low index values compared to RG. This indicates a higher burden of morbidity in the patient sample in PCNs. The percentage of included cases who needed extended care in a nursing home was 0.6% in PCNs (n = 116) and 0.4% in RG (n = 13,513). Table 3 details health status across all cases regarding cases included in Disease Management Programs (DMP), nursing care level, and Charlson co-morbidity index. (See Additional file 2 for detailed information on CCI per observed infection and respective patient population.)

**Table 3:** Distribution of disease management programs and health status across all cases

Health status	RG	PCN
Disease Management Program (DMP): n (%)	Diabetes Type 2: 2,140,035 (68.8)	Diabetes Type 2: 16,754 (92.0)
	Asthma: 2,388,555 (76.8)	Asthma: 16,913 (92.9)
	COPD: 2,042,731 (65.7)	COPD: 16,805 (92.3)
	Coronary heart disease: 2,107,723 (67.7)	Coronary heart disease: 16,767 (92.1)
Nursing care Level*: n (%)	1: 1530 (0.0) 2: 31,036 (1.0) 3: 19,713 (0.6) 4: 10,966 (0.4) 5: 4011 (0.1)	1: 23 (0.1) 2: 272 (1.5) 3: 164 (0.9) 4: 65 (0.4) 5: 19 (0.1)
Charlson Index: n (%)	0: 2,178,429 (70.0) 1, 2: 757,952 (24.4) 3, 4: 105,140 (3.4) > 5: 69,561 (2.2)	0: 10,059 (55.2) 1, 2: 5482 (30.1) 3, 4: 1334 (7.3) > 5: 1332 (7.3)

*The nursing care level reflects the extent to which patients are able to manage their own needs independently. Based on an expected care dependency of at least 6 months, evaluation takes six main aspects into account: mobility, cognitive and communicative abilities, behavioral and psychological issues, self-care, management of disease-related demands and burden, and arrangements of daily life and social contacts [21]

### Secondary outcomes

Regression analysis for prescribing of guideline-recommended antibiotics did not identify common influencing factors over all considered infections (see Additional file 2). Pediatricians seemed to prescribe more recommended antibiotics compared to general practitioners for patients suffering from URTI. For bronchitis, older patients (> 65 years) had a lower probability to receive a recommended antibiotic prescription compared to patients with age 18–65. Detailed results regarding prescribing of guideline-recommended antibiotics (1st choice and alternatives) across all observed cases in PCNs and RG are given in Table 4. Listed values represent prescribing of one total year.

**Table 4:** Prescribing of guideline-recommended antibiotics across all cases

	RG cases 1st choice/ alternative choice/ total	PCN cases 1st choice/ alternative choice/ total
Tonsillitis %*	24.0/ 3.8 / 27.8	18.8/ 1.2 / 20
Sinusitis %	18.7/ 40.6 / 59.3	22.1/ 42.2 / 64.3
Otitis media %	41.0/ 32.4 / 73.4	28.6/ 37.5 / 66.1
Bronchitis %	18.5/ 43.8 / 62.3	23.1/ 38.6 / 61.7
Upper respiratory tract infections %	22.1/**	18.5/**

*RG*  reference group representing standard care, *PCN*  primary care network

*Diagnoses for streptococcal tonsilitis and other pathogen-caused acute forms of tonsillitis that warrant antibiotic therapy are not included

**No alternative choices defined

Overall, quinolones were prescribed in 9.9% of cases in PCNs and 8.1% in RG and thus, generally moderate to low. In PCNs, prescribing of quinolones was observed in 11.3% of cases with bronchitis and 9.5% of the cases with URTI (in RG 11.4% and 7.7%). Viewed separately, 4.9% of Otitis Media cases, 3.6% of Tonsillitis cases, and 11.2% of Sinusitis cases received a prescription for quinolones in PCNs. With 5.3%, 2.7%, and 9.5%, the respective proportions of quinolone prescribing were somewhat different in RG. In mixed logistic regression models for quinolone prescribing for patients treated with antibiotics in PCNs, patients in increasingly urbanized areas seemed to be less likely to receive a prescription for quinolones. An increased CCI implied higher probability for prescribing quinolones compared to a CCI of 0 (no comorbidity). Detailed results of the logistics mixed effects model are given in Table 5. (See Additional file 1: Table S4 for diagnoses that warrant quinolone prescribing.)

**Table 5:** Results of the logistic mixed effects regression model for prescribing rates of quinolones for patients treated with antibiotics in PCNs

Covariate	OR	Lower CI limit	Upper CI limit	Standard error	p-value
Other spec. groups vs.* General Practitioner	0.617	0.277	1.374	0.408	0.237
Urbanization vs.* rural location	0.466	0.210	1.035	0.407	0.061
Urban vs. rural	0.558	0.371	0.840	0.209	0.005
PCN size medium vs*. small	1.975	1.028	3.793	0.333	0.041
PCN size large vs.* small	1.077	0.565	2.053	0.329	0.821
Patient age > 65 vs.* age < = 65	1.294	0.997	1.679	0.133	0.053
Female patients vs.* Male patients	0.928	0.757	1.136	0.104	0.467
Patients with Charlson Index 1 and 2 vs.*0	1.864	1.469	2.366	0.122	< 0.001
Patients with Charlson Index 3 and 4 vs.* 0	3.114	2.196	4.418	0.178	< 0.001
Patients with Charlson Index > = 5 vs.* 0	3.264	2.245	4.746	0.191	< 0.001

*vs*  versus, *PCN*  primary care network

On practice level, the prescribing of broad-spectrum antibiotics in DDD% were higher in PCNs (Median [Q1–Q3]: 100% [82.4–100]) compared to RG (Median [Q1–Q3]: 95.9% [66.1–100]). Beta regression models on practice level indicated that specialist groups had a lower prescribing rate of broad-spectrum antibiotics than GPs. (See Additional file 2: Table S17).

The descriptive subgroup analyses over all patients (PCNs and RG) support the findings of the regression models. Detailed results of the descriptive subgroups analysis corresponding to the primary and secondary outcomes are shown in Additional file 2. The rates of guideline-recommended substances were comparable between genders. Contrasting the participation in DMPs resulted in a small increase in the rate of antibiotics and quinolones for DMP participants, and smaller rates of recommended (alternative) antibiotics for indexed diagnoses. A clear difference is observed for CCI 0 (without comorbidity) versus higher values. Antibiotics and quinolone prescribing rates were more than 10% higher for patients with comorbidities and less recommended substances were used for those patients.

## Discussion

This study explored baseline antibiotic prescribing rates in PCNs at the start of the ARena project regarding cases with acute non-complicated respiratory tract infections. In about a third of all observed cases, patients received an antibiotic. Younger patients were less likely to receive antibiotics. More antibiotics were prescribed to female than to male patients. GPs were the largest included group of prescribers and prescribed antibiotics more frequently than other medical specialists. Prescribing rates for quinolones were moderate, and patients with comorbidities had a higher likelihood of receiving an antibiotic and quinolone prescription and less likely to receive a guideline-recommended substance.

Rates of antibiotic prescribing in German primary care overall have been decreasing constantly between 2010 and 2018, particularly with regards to children and adolescents [22] who were underrepresented in this study sample. Room for improvement can be expected to be more visible in ‘high prescribers’, but since the potential for improvement noticeably decreased in recent years already, nevertheless, there remains substantial room for further reduction of antibiotic prescribing.

A recent national cross-sectional study in USA identified that 57% of 130.5 million prescriptions for antibiotics written during ambulatory care visits in 2015 were for appropriate indications, 25% were inappropriate and 18% had no documented indication. It was noted that being an adult male, spending more time with the provider and seeing a non-primary care specialist were significantly positively associated with non-indicated antibiotic [23]. In contrast to these findings, our data indicate that antibiotics were most frequently prescribed by general practitioners. In addition, the prescribing of broad-spectrum antibiotics showed slightly higher rates in PCNs compared to RG. These discrepancies in antibiotic prescribing rates between PCNs and RG might be explained by the differing specialist group distribution between PCNs and RG and a small percentage of specialists and a higher percentage of GPs in PCNs than RG. A contributing factor towards the slight difference in prescribing rates between PCNs and RG can also be seen in higher patient age and morbidity in PCNs which is in line with findings of Shaver et al. [24] who recently examined antibiotic prescribing in the outpatient setting in USA. Exception here in our study is prescribing for Otitis media where cases were included from the age of two and above and therefore morbidity considerations cannot deliver explanations for higher prescribing rates in PCNs. Prescribing rates for quinolones were generally moderate to low in PCNs (9.9%) and RG (8.1%) and thus also match findings of the study from USA where in 9.4% of investigated visits for acute respiratory infections a broad-spectrum antibiotic was received [24]. Prescribing quinolones seemed to be associated with a higher number of comorbidities and higher patient age as well which complements findings of the process evaluation conducted alongside ARena where uncertainty about diagnoses, prognosis, continuity of care and perceptions about patient preferences were found to be among the reasons for non-indicated prescribing [25, 26]. To some extend this confirms findings from a 2010 study that investigated fluoroquinolone prescribing for acute cough in German primary care to find predictors for unjustified prescribing. The researchers then concluded that unjustified quinolone prescribing was determined by patient characteristics such as severity and duration of illness and patient age. However, they also found that physicians with higher individual antibiotic prescribing rates and physicians with hospital-based specialty training—versus combined hospital and ambulatory training—were more likely to prescribe fluoroquinolones than physicians who specifically trained as GPs [27]. Interestingly, our data showed that in PCN practices in locations with increasing urbanization, quinolone prescribing rates were lower than in rural locations. This complements results from a previous study where German GPs working in urban areas were found to be more likely to use the strategy of delayed prescribing of antibiotics than GPs working in rural areas [11]. Such discrepancies between urban and rural prescribing habits potentially could root in more ample opportunities for self-reflection about prescribing motivated by regular peer exchange and a broader and more frequent offer of continued training and upskilling in urban locations. Efforts to educate physicians, care teams and patients continuously about current and appropriate diagnosis and therapy options as well as communication about them may remedy this to some extent.

The perceived, but not actually communicated patient request for antibiotics is often overrated by physicians [28] and may initiate unfounded assumptions of losing patients to another physician when therapy followed guideline recommendations and antibiotics are not prescribed. In this study, the rate for prescribing of guideline-recommended antibiotics was remarkably low and in line with the high rate for broad-spectrum antibiotics on practice level which is another potential indicator for physicians’ uncertainty. Though quinolone prescribing was moderate overall, patients with higher comorbidity and patients participating in DMPs were more likely to receive a quinolone prescription, and less likely to receive one of the guideline-recommended substances. This might be related to higher age and poorer health status of these patients as supported by previous research [24], but also to the physicians’ striving for the elimination of both their own and patients’ insecurities and potential complications. One effective way to eliminate insecurities and reduce prescribing of antibiotics was found to be the promotion of communication skills by means of a short communication training for primary care physicians [29, 30]. In a recent study in Germany, results showed a prescribing probability decrease of 6.5 percentage points for the treatment of URTI and an even stronger impact for female patients aged below 35 [31]. As the ARena project also used an intervention component to strengthen communication skills, similar effects can be expected.

The rate of prescribed broad-spectrum antibiotics in PCNs in this study seemed slightly higher compared to the study by Shaver et al. conducted in USA [24]. However, for this present study the outcome was assessed by DDD% on practice level whereas Shaver et al. evaluated on patient level. Factors associated with the prescribing of guideline-recommended antibiotics for the considered infections could not be clearly identified. Information about patient nationality was not available for all cases, yet the percentage of nationalities other than German was low. However, our data showed that the percentage of observed cases of Eastern European patients was higher than the one of Southern Europeans in PCNs as well as in RG. This is contrasted by findings of the process evaluation in ARena where physicians indicated their subjective perception of many Southern Europeans asking for antibiotics [25].

### Strengths and limitations

One strength of this study is the careful exclusion of cases where there was an ICD-10 code documented that indicates antibiotic prescribing. Additionally, the type of antibiotics (recommended, quinolones, broad- versus small-spectrum) were analyzed in detail. A limitation of this retrospective cross-sectoral analysis of baseline data is the restriction on ICD-10 codes and claims-related health insurance data. Therefore disease, patient, and practice information are limited. Direct connection between ICD-10 code and prescription of antibiotics is not possible in the provided claims data, thus ICD-10 codes and antibiotics prescription were matched by quarter which introduces a potential bias. To compensate, diagnoses that warranted antibiotic therapy were excluded. The use of DDDs for pediatric antibiotic consumption where weight-based dosing is appropriate, is an additional limitation. Participating practices and PCNs were already recruited for the ARena trial and might be more alert to the topic and supportive of appropriate prescribing already (shared attitude in the networks that might only be attractive for certain physicians). So far only one time point before the planned intervention in the ARena trial could be analyzed. Final assessment of effects will be possible through the analysis of post intervention data with a focus on the evaluation of the interventions. The baseline data included patients and practices as clustering levels. Since only a small number of patients produced more than one case, patients were not considered as random intercept in the model. P-values are of secondary interest since the population considered here is very large and p-values get very small even for small differences. Findings of this present study can then serve as comparator in the final report of the outcome evaluation.

## Conclusion

Primary care antibiotic prescribing rates for acute non-complicated respiratory tract infections were moderate prior to the intervention start of the ARena project, but indicate room for improvement. Lower rates for guideline-recommended substances indicate a need for creating stronger awareness of guideline-conform use of antibiotics as intended by the implementation program used in ARena. This baseline assessment of prescribing will serve as comparator in the post-interventional outcome evaluation and support describing of potentially observed effects of the program.

## Supplementary Information

**Additional file 1: Table S1.** Diagnoses Primary Outcome. **Table S2.** Excluded Diagnoses. **Table S3.** Recommended Antibiotics. **Table S4.** Diagnoses for Quinolone Prescription. **Table S5.** Distribution of medical specialty per infection treated with antibiotics.

**Additional file 2: Table S6.** Recommended Antibiotics for Otitis Media. **Table S7.** Logistics mixed effects model for the rate of recommended antibiotics for patients with Otitis Media and antibiotics prescription for practices participating in ARena. The practices are considered as random effect. Documented are odds ratios (OR), corresponding confidence interval (CI) limits, standard errors, and p-value. **Table S8. **Recommended Antibiotics for Tonsillitis. **Table S9.** Logistics mixed effects model for the rate of recommended antibiotics for patients with Tonsillitis and antibiotics prescription for practices participating in ARena. The practices are considered as random effect. Documented are odds ratios (OR), corresponding confidence interval (CI) limits, standard errors, and p-value. **Table S10. **Recommended Antibiotics Sinusitis**. Table S11.** Logistics mixed effects model for the rate of recommended antibiotics for patients with Sinusitis and antibiotics prescription. The practices are considered as random effect. Documented are odds ratios (OR), corresponding confidence interval (CI) limits, standard errors, and p-value. **Table S12.** Recommended Antibiotics Bronchitis**. Table S13.** Logistics mixed effects model for the rate of recommended antibiotics for patients with Bronchitis and antibiotics prescription. The practices are considered as random effect. Documented are odds ratios (OR), corresponding confidence interval (CI) limits, standard errors, and p-value. **Table S14.** Recommended Antibiotics Upper Respiratory Infections. Patient and health status characteristics for patients suffering from upper respiratory infection with antibiotics prescription. **Table S15.** Logistics mixed effects model for the rate of recommended antibiotics for patients with Upper Respiratory Infections and antibiotics prescription. The practices are considered as random effect. Documented are odds ratios (OR), the corresponding confidence interval (CI) limits, standard errors, and p-value. **Table S16. **Quinolones**. Table S17.** Broad-spectrum antibiotics. **Table S18.** Primary and secondary outcomes contrasted for gender. **Table S19**. Primary and secondary outcomes contrasted for DMP. **Table 20.** Primary and secondary outcomes contrasted for Charlson Index.

## Data Availability

All analyses generated for this study are included in this published article and its Additional information files 1 and 2. The original datasets that support the findings of this study are not publicly available due to restrictions stipulated by German law and the data provider. AOK can be contacted for access to the data.

## Abbreviations

ARTI: Acute respiratory tract infections
AWMF: Association of the Scientific Medical Societies in Germany
CAP: Community acquired pneumonia
CI: Confidence interval
CCI: Charlson comorbidity Index
DDD: Defined daily dose
DEGAM: German College of General Practitioners and Family Physicians
DMP: Disease management program
ESAC-Net: European Surveillance of Antimicrobial Consumption Network
GP: General practitioners
ICD-10: International Classification of Diseases
OR: Odds ratio
PCNs: Primary care networks
Q: Quarter
RG: Reference group reflecting standard care
URTI: Upper respiratory tract infections
