# Supplementary material for: Antibiotic prescribing for acute, non-complicated infections in primary care in Germany: baseline assessment in the cluster randomized trial ARena
Source: BMC Infect Dis. 2021 Aug 26;21:877. doi: 10.1186/s12879-021-06571-0 (PMC8394572; doi:10.1186/s12879-021-06571-0)
Supplement: Supplementary file 1 — Additional file 1: Table S1. Diagnoses Primary Outcome. Table S2. Excluded Diagnoses. Table S3. Recommended Antibiotics. Table S4. Diagnoses for Quinolone Prescription. Table S5. Distribution of medical specialty per infection treated with antibiotics. [file 12879_2021_6571_MOESM1_ESM.docx]

**Additional file 1**

**Supplementary Table 1: Diagnoses Primary Outcome**

| Diagnoses | ICD Code |
| --- | --- |
| Upper Respiratory Infections | J00; J02.0; J02.8, J02.9; J04; J06; J10.1; J11.1 |
| Bronchitis | J20; J21.0; J21.1, J21.8; J21.9; J22; J40 |
| Tonsillitis | J03.9 |
| Sinusitis | J01 |
| Otitis Media | H65.0; H65.1; H65.9; H66.0; H66.4; H66.9 |

**Supplementary Table 2: Excluded Diagnoses***

|  |  |
| --- | --- |
| A00 bis A79.9 | infectious colon diseases;  [[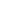](https://www.icd-code.de/icd/code/A00-A09.html)](https://www.icd-code.de/icd/code/A00-A09.html)Tuberculosis; specific bacterial zoonoses; other bacterial diseases; [[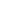](https://www.icd-code.de/icd/code/A30-A49.html)](https://www.icd-code.de/icd/code/A30-A49.html)sexually transmitted diseases; other spirochetes diseases; other chlamydia-induced diseases; [[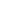](https://www.icd-code.de/icd/code/A70-A74.html)](https://www.icd-code.de/icd/code/A70-A74.html)Rickettsioses |
| E84 | Cystic fibrosis |
| J44.0, J44.1 | Other chronic obstructive lung diseases |
| N10 | Acute tubulo-interstitial nephritis |
| N12 | Nephritis not acute or interstitial |
| N30 | Cystitis |
| N39.0 | Other urinary tract infections |
| N41 | Inflammatory prostate diseases |
| O | Pregnancy, birth, puerperium |

*Cases with excluded diagnoses in an observed quarter are not considered for analysis.

**Supplementary Table 3: Recommended Antibiotics**

|  | Recommended Antibiotics | Alternative Antibiotics |
| --- | --- | --- |
| Upper Respiratory Infections | J01CA04 (Amoxicillin) | - |
| Bronchitis | J01CA04 (Amoxicillin) | J01AA02 (Doxycyclin); J01CE02 (Phenoxymethylpenicillin); J01FA (Macrolides) |
| Tonsillitis | J01CE02 (Phenoxymethylpenicillin) | J01DB (Cephalosporins); J01FA01(Erythromycin) |
| Sinusitis | J01CA04 (Amoxicillin) | J01DC (Cephalosporins 2^nd^ Gen); J01AA02 (Doxycyclin); J01EE01 (Sulfamethoxazol and Trimethoprim); J01CR02 (Amoxicillin and beta-lactamase-inhibitors); J01CR22 (Amoxicillin and Clavulanic acid) |
| Otitis Media | J01CA04 (Amoxicillin) | J01DC (Cephalosporins 2^nd^ Gen); J01FA01 (Erythromycin); J01CR02 (Amoxicillin and beta-lactamase-inhibitors); J01CR22 (Amoxicillin and Clavulanic acid) |

**Supplementary Table 4: Diagnoses for Quinolone Prescription**

| Upper Respiratory Infections | J00; J02.0; J02.8, J02.9; J04; J06; J10.1; J11.1 |
| --- | --- |
| Bronchitis | J20; J21.0; J21.1, J21.8; J21.9; J22; J40 |
| Tonsillitis | J03.0; J03.8; J03.9 |
| Sinusitis | J01 |
| Otitis Media | H65.0; H65.1; H65.9; H66.0; H66.4; H66.9 |

**Supplementary Table 5: Distribution of medical specialty per infection treated with antibiotics**

| **Observed infection** | **Treating physician** | **RGs** | **PCNs** |
| --- | --- | --- | --- |
| Otitis media | General Practitioner | 43 185 (46.9%) | 247 (87.3%) |
|  | Otolaryngologist | 21 693 (23.6%) | 20 (7.1%) |
|  | Pediatrician | 26 540 (28.9%) | 16 (5.7%) |
|  | Other | 567 (0.6%) | 0(0%) |
| Sinusitis | General Practitioner | 73 209 (71.3%) | 691 (92.4%) |
|  | Otolaryngologist | 28 647 (27.9%) | 56 (7.5%) |
|  | Other | 750 (0.7%) | 1 (0.1%) |
| Tonsillitis | General Practitioner | 150 374 (65.9%) | 742 (89.5%) |
|  | Otolaryngologist | 21 001 (9.2%) | 43 (5.2%) |
|  | Pediatrician | 54 851 (24.1%) | 44 (5.3%) |
|  | Other | 1 826 (0.8%) | 0 (0%) |
| Bronchitis | General Practitioner | 319 810 (96.7%) | 2 534 (99.7%) |
|  | Other | 10 744 (3.3%) | 8 (0.3%) |
| Upper resp. infection | General Practitioner | 390 839 (76.6%) | 2 883 (96.3%) |
|  | Otolaryngologist | 65 828 (12.9%) | 84 (2.8%) |
|  | Pediatrician | 49 425 (9.7%) | 26 (0.9%) |
|  | Other | 4 070 (0.8%) | 1 (0.0%) |
| acute non-complicated infections (quinolones) | General Practitioner | 799 194 (77.7%) | 5 761 (96.0%) |
|  | Otolaryngologist | 106 649 (10.4%) | 155 (2.6%) |
|  | Pediatrician | 110 278 (10.7%) | 77 (1.3%) |
|  | Other | 11 902 (1.2%) | 5 (0.1%) |
