# Supplementary material for: Antibiotic prescribing for acute, non-complicated infections in primary care in Germany: baseline assessment in the cluster randomized trial ARena
Source: BMC Infect Dis. 2021 Aug 26;21:877. doi: 10.1186/s12879-021-06571-0 (PMC8394572; doi:10.1186/s12879-021-06571-0)
Supplement: Supplementary file 2 — Additional file 2: Table S6. Recommended Antibiotics for Otitis Media. Table S7. Logistics mixed effects model for the rate of recommended antibiotics for patients with Otitis Media and antibiotics prescription for practices participating in ARena. The practices are considered as random effect. Documented are odds ratios (OR), corresponding confidence interval (CI) limits, standard errors, and p-value. Table S8. Recommended Antibiotics for Tonsillitis. Table S9. Logistics mixed effects model for the rate of recommended antibiotics for patients with Tonsillitis and antibiotics prescription for practices participating in ARena. The practices are considered as random effect. Documented are odds ratios (OR), corresponding confidence interval (CI) limits, standard errors, and p-value. Table S10. Recommended Antibiotics Sinusitis. Table S11. Logistics mixed effects model for the rate of recommended antibiotics for patients with Sinusitis and antibiotics prescription. The practices are considered as random effect. Documented are odds ratios (OR), corresponding confidence interval (CI) limits, standard errors, and p-value. Table S12. Recommended Antibiotics Bronchitis. Table S13. Logistics mixed effects model for the rate of recommended antibiotics for patients with Bronchitis and antibiotics prescription. The practices are considered as random effect. Documented are odds ratios (OR), corresponding confidence interval (CI) limits, standard errors, and p-value. Table S14. Recommended Antibiotics Upper Respiratory Infections. Patient and health status characteristics for patients suffering from upper respiratory infection with antibiotics prescription. Table S15. Logistics mixed effects model for the rate of recommended antibiotics for patients with Upper Respiratory Infections and antibiotics prescription. The practices are considered as random effect. Documented are odds ratios (OR), the corresponding confidence interval (CI) limits, standard errors, and p-value. Table S16. [file 12879_2021_6571_MOESM2_ESM.docx]

**Additional file 2**

**Supplementary Table 6: Recommended Antibiotics for Otitis Media**

Patient and health status characteristics for patients with Otitis Media and antibiotics prescription

|  | RGs (n= 91 985) | PCNs (n=283) |
| --- | --- | --- |
| Age |  |  |
| Mean (SD) | 24.2 (21.02) | 42.8 (20.69) |
| Median [Q1-Q3] | 17 [6 – 39] | 43 [27 – 59] |
| Min. -- Max. | 3.0 - 105.0 | 3.0 - 93.0 |
| Age categories |  |  |
| <18 | 46 373 (50.4%) | 35 (12.4%) |
| 18-65 | 41 364 (45.0%) | 206 (72.8%) |
| >65 | 4 248 (4.6%) | 42 (14.8%) |
| Gender |  |  |
| male | 42 734 (46.5%) | 96 (33.9%) |
| female | 49 251 (53.5%) | 187 (66.1%) |
| Charlson Index (categories) |  |  |
| 0 | 72 569 (78.9%) | 175 (61.8%) |
| 1, 2 | 16 311 (17.7%) | 77 (27.2%) |
| 3, 4 | 1 847 (2.0%) | 15 (5.3%) |
| >=5 | 1 258 (1.4%) | 16 (5.7%) |

**Supplementary Table 7:** Logistics mixed effects model for the rate of recommended antibiotics for patients with Otitis Media and antibiotics prescription for practices participating in ARena. The practices are considered as random effect. Documented are odds ratios (OR), corresponding confidence interval (CI) limits, standard errors, and p-value.

| Covariate | OR | Lower CI limit | Upper CI limit | St. error | p-value |
| --- | --- | --- | --- | --- | --- |
| Other spec. groups vs. General Practitioner | 3.380 | 0.965 | 11.836 | 0.640 | 0.057 |
| Urbanization vs. rural location | 3.784 | 0.672 | 21.325 | 0.882 | 0.131 |
| Urban vs. rural location | 1.216 | 0.466 | 3.174 | 0.489 | 0.689 |
| PCN size medium vs. small | 0.942 | 0.222 | 3.993 | 0.737 | 0.935 |
| PCN size large vs. small | 1.096 | 0.284 | 4.236 | 0.690 | 0.894 |
| Patient age <18 vs. age 18-65 | 1.486 | 0.498 | 4.432 | 0.558 | 0.478 |
| Patient age >65 vs. age 18-65 | 0.792 | 0.254 | 2.470 | 0.580 | 0.688 |
| Female patients vs. male | 1.432 | 0.702 | 2.919 | 0.364 | 0.324 |
| Charlson Index 1, 2 vs. 0 | 0.945 | 0.441 | 2.023 | 0.389 | 0.883 |
| Charlson Index 3, 4 vs. 0 | 0.332 | 0.034 | 3.242 | 1.163 | 0.343 |
| Charlson Index >=5 vs. 0 | 0.323 | 0.050 | 2.075 | 0.949 | 0.234 |

**Supplementary Table 8: Recommended Antibiotics for Tonsillitis**

Patient and health status characteristics for patients suffering from Tonsillitis with antibiotics prescription.

|  | RGs (n= 228 052) | PCNs (n= 829) |
| --- | --- | --- |
| Age |  |  |
| Mean (SD) | 24.4(17.40) | 36.9 (18.33) |
| Median [Q1-Q3] | 21 [9-35] | 35 [22-50] |
| Min. -- Max. | 2.0 - 102.0 | 2.0 - 100.0 |
| Age (categories) |  |  |
| <18 | 94 589 (41.5%) | 115 (13.9%) |
| 18-65 | 128 429 (56.3%) | 656 (79.1%) |
| >65 | 5 034 (2.2%) | 58 (7.0%) |
| Gender |  |  |
| Male | 103 515 (45.4%) | 323 (39.0%) |
| Female | 124 537 (54.6%) | 506 (61.0%) |
| Charlson Index (categories) |  |  |
| 0 | 189 064 (82.9%) | 593 (71.5%) |
| 1, 2 | 34 646 (15.2%) | 172 (20.7%) |
| 3, 4 | 2 662 (1.2%) | 29 (3.5%) |
| >= 5 | 1 680 (0.7%) | 35 (4.2%) |

**Supplementary Table 9:** Logistics mixed effects model for the rate of recommended antibiotics for patients with Tonsillitis and antibiotics prescription for practices participating in ARena. The practices are considered as random effect. Documented are odds ratios (OR), corresponding confidence interval (CI) limits, standard errors, and p-value.

| Covariate | OR | Lower CI limit | Upper CI limit | St. error | p-value |
| --- | --- | --- | --- | --- | --- |
| Otolaryngologist vs. General Practitioner | 0.363 | 0.051 | 2.595 | 1.004 | 0.312 |
| Pediatrician vs. General Practitioner | 0.903 | 0.050 | 16.458 | 1.481 | 0.945 |
| Urbanization vs. rural location | 1.793 | 0.223 | 14.411 | 1.063 | 0.583 |
| Urban vs. rural location | 1.624 | 0.544 | 4.854 | 0.559 | 0.385 |
| PCN size medium vs. small | 0.112 | 0.019 | 0.646 | 0.894 | 0.014 |
| PCN size large vs. small | 0.474 | 0.096 | 2.339 | 0.814 | 0.359 |
| Patient age <18 vs. age 18-65 | 1.487 | 0.653 | 3.387 | 0.420 | 0.345 |
| Patient age >65 vs. age 18-65 | 1.043 | 0.344 | 3.160 | 0.566 | 0.941 |
| Female patients vs. male | 1.330 | 0.814 | 2.171 | 0.250 | 0.255 |
| Charlson Index 1, 2 vs. 0 | 0.746 | 0.392 | 1.417 | 0.328 | 0.370 |
| Charlson Index 3, 4 vs. 0 | 0.546 | 0.122 | 2.446 | 0.765 | 0.429 |
| Charlson Index >=5 vs. 0 | 0.362 | 0.068 | 1.936 | 0.856 | 0.235 |

**Supplementary Table 10: Recommended Antibiotics Sinusitis**

Patient and health status characteristics for patients suffering from Sinusitis with antibiotics prescription.

|  | RGs (n= 102 606) | PCNs (n=748) |
| --- | --- | --- |
| Age |  |  |
| Mean (SD) | 43.8 (15.75) | 50.0 (16.19) |
| Median [Q1-Q3] | 43 [31-55] | 50 [37-60] |
| Min. - Max. | 19.0 - 101.0 | 19.0 - 94.0 |
| Age (categories) |  |  |
| 18-65 | 92 915 (90.6%) | 612 (81.8%) |
| >65 | 9 691 (9.4%) | 136 (18.2%) |
| Gender |  |  |
| Male | 34 244 (33.4%) | 231 (30.9%) |
| Female | 68 362 (66.6%) | 517 (69.1%) |
| Charlson Index (categories) |  |  |
| 0 | 69 504 (67.7%) | 431 (57.6%) |
| 1, 2 | 26 520 (25.8%) | 209 (27.9%) |
| 3, 4 | 4 053 (4.0%) | 54 (7.2%) |
| >= 5 | 2 529 (2.5%) | 54 (7.2%) |

**Supplementary Table 11:** Logistics mixed effects model for the rate of recommended antibiotics for patients with Sinusitis and antibiotics prescription. The practices are considered as random effect. Documented are odds ratios (OR), corresponding confidence interval (CI) limits, standard errors, and p-value.

| Covariate | OR | Lower CI limit | Upper CI limit | St. error | p-value |
| --- | --- | --- | --- | --- | --- |
| Otolaryngologist vs. General Practitioner | 1.228 | 0.418 | 3.607 | 0.550 | 0.709 |
| Urbanization vs. rural location | 1.090 | 0.280 | 4.253 | 0.695 | 0.901 |
| Urban vs. rural | 1.585 | 0.714 | 3.518 | 0.407 | 0.257 |
| PCN size medium vs. small | 1.479 | 0.474 | 4.613 | 0.580 | 0.500 |
| PCN size large vs. small | 0.973 | 0.323 | 2.930 | 0.562 | 0.962 |
| Patient age >65 vs. age 18-65 | 0.945 | 0.521 | 1.715 | 0.304 | 0.853 |
| Female patients vs. male | 0.691 | 0.449 | 1.063 | 0.220 | 0.093 |
| Charlson Index 1, 2 vs. 0 | 0.967 | 0.587 | 1.593 | 0.255 | 0.894 |
| Charlson Index 3, 4 vs. 0 | 1.348 | 0.607 | 2.995 | 0.407 | 0.463 |
| Charlson Index >=5 vs. 0 | 1.167 | 0.487 | 2.795 | 0.446 | 0.729 |

**Supplementary Table 12: Recommended Antibiotics Bronchitis**

Patient and health status characteristics for patients suffering from Bronchitis with antibiotics prescription.

|  | RGs (n=330 554) | PCNs (n=2 542) |
| --- | --- | --- |
| Age |  |  |
| Mean (SD) | 46.6 (14.74) | 52.3 (13.66) |
| Median [Q1-Q3] | 48 [34 - 58] | 54 [44 - 62] |
| Min. -- Max. | 19.0 - 74.0 | 19.0 - 74.0 |
| Age (categories) |  |  |
| 18-65 | 294 700 (89.2%) | 2 095 (82.4%) |
| >65 | 35 854 (10.8%) | 447 (17.6%) |
| Gender |  |  |
| Male | 150 257 (45.5%) | 1 016 (40.0%) |
| Female | 180 297 (54.5%) | 1 526 (60.0%) |
| Charlson Index (categories) |  |  |
| 0 | 120 642 (36.5%) | 897 (35.3%) |
| 1, 2 | 175 597 (53.1%) | 1 172 (46.1%) |
| 3, 4 | 21 615 (6.5%) | 248 (9.8%) |
| >= 5 | 12 700 (3.8%) | 225 (8.9%) |

**Supplementary Table 13:** Logistics mixed effects model for the rate of recommended antibiotics for patients with Bronchitis and antibiotics prescription. The practices are considered as random effect. Documented are odds ratios (OR), corresponding confidence interval (CI) limits, standard errors, and p-value.

| Covariate | OR | Lower CI limit | Upper CI limit | St. error | p-value |
| --- | --- | --- | --- | --- | --- |
| Other spec. groups vs. General Practitioner | 5.751 | 0.807 | 41.012 | 1.002 | 0.081 |
| Urbanization vs. rural | 3.235 | 1.008 | 10.385 | 0.595 | 0.049 |
| Urban vs. rural | 0.893 | 0.484 | 1.647 | 0.313 | 0.717 |
| PCN size medium vs. small | 1.838 | 0.685 | 4.931 | 0.504 | 0.227 |
| PCN size large vs. small | 1.704 | 0.641 | 4.526 | 0.499 | 0.285 |
| Patient age >65 vs. age 18-65 | 0.659 | 0.477 | 0.911 | 0.165 | 0.012 |
| Female patient vs. male | 0.933 | 0.747 | 1.165 | 0.113 | 0.539 |
| Charlson Index 1, 2 vs. 0 | 0.868 | 0.666 | 1.130 | 0.135 | 0.293 |
| Charlson Index 3, 4 vs. 0 | 0.894 | 0.595 | 1.344 | 0.208 | 0.591 |
| Charlson Index >=5 vs. 0 | 0.817 | 0.519 | 1.287 | 0.232 | 0.384 |

**Supplementary Table 14: Recommended Antibiotics Upper Respiratory Infections**

Patient and health status characteristics for patients suffering from upper respiratory infection with antibiotics prescription.

|  | RGs (n= 510 162) | PCNs (n= 2 994 ) |
| --- | --- | --- |
| Age |  |  |
| Mean | 37.3 (21.27) | 49.3 (18.60) |
| Median [Q1-Q3] | 36 [20 - 53] | 50 [37 - 61] |
| Min. - Max. | 2.0 - 106.0 | 2.0 - 100.0 |
| Age (categories) |  |  |
| <18 | 104 204 (20.4%) | 158 (5.3%) |
| 18-65 | 355 213 (69.6%) | 2 285 (76.3%) |
| >65 | 50 745 (9.9%) | 551 (18.4%) |
| Gender |  |  |
| Male | 223 002 (43.7%) | 1 116 (37.3%) |
| Female | 287 160 (56.3%) | 1 878 (62.7%) |
| Charlson Index (categories) |  |  |
| 0 | 342 048 (67.0%) | 1 554 (51.9%) |
| 1, 2 | 133 168 (26.1%) | 966 (32.3%) |
| 3, 4 | 20 600 (4.0%) | 236 (7.9%) |
| >= 5 | 14 346 (2.8%) | 238 (7.9%) |

**Supplementary Table 15:** Logistics mixed effects model for the rate of recommended antibiotics for patients with Upper Respiratory Infections and antibiotics prescription. The practices are considered as random effect. Documented are odds ratios (OR), the corresponding confidence interval (CI) limits, standard errors, and p-value.

| Covariate | OR | Lower CI limit | Upper CI limit | St. error | p-value |
| --- | --- | --- | --- | --- | --- |
| Otolaryngologist vs. General Practitioner | 1.287 | 0.470 | 3.524 | 0.514 | 0.623 |
| Pediatrician vs. General Practitioner | 6.274 | 0.738 7 | 53.347 | 1.092 | 0.093 |
| Urbanization vs. Rural location | 1.647 | 0.572 | 4.741 | 0.540 | 0.355 |
| Urban vs. rural location | 1.105 | 0.615 | 1.984 | 0.299 | 0.739 |
| PCN size medium vs. small | 1.185 | 0.475 | 2.958 | 0.467 | 0.715 |
| PCN size large vs. small | 1.236 | 0.503 | 3.036 | 0.459 | 0.644 |
| Patient age <18 vs. age 18-65 | 1.204 | 0.747 | 1.941 | 0.244 | 0.447 |
| Patient alter >65 vs. age 18-65 | 1.040 | 0.747 | 1.449 | 0.169 | 0.816 |
| Female patients vs. male | 0.842 | 0.674 | 1.051 | 0.113 | 0.128 |
| Charlson Index 1, 2 vs. 0 | 0.795 | 0.616 | 1.024 | 0.130 | 0.076 |
| Charlson Index 3, 4 vs. 0 | 0.809 | 0.511 | 1.282 | 0.235 | 0.367 |
| Charlson Index >=5 vs. 0 | 0.711 | 0.440 | 1.148 | 0.244 | 0.163 |

**Supplementary Table 16: Quinolones**

Patient and health status characteristics for patients suffering from acute non-complicated infections with antibiotics prescription.

|  | RGs (n= 1 028 023) | PCNs (n= 5 998) |
| --- | --- | --- |
| Age |  |  |
| Mean | 37.0 (20.75) | 49.2 (17.91) |
| Median [Q1-Q3] | 36 [21 - 53] | 51 [37 - 61] |
| Min. - Max. | 2.0 - 106.0 | 2.0 - 100.0 |
| Age (categories) |  |  |
| <18 | 208 748 (20.3%) | 278 (4.6%) |
| 18-65 | 727 101 (70.7%) | 4 637 (77.3%) |
| >65 | 92 174 (9.0%) | 1 083 (18.1%) |
| Gender |  |  |
| Male | 456 195 (44.4%) | 2 295 (38.3%) |
| Female | 571 828 (55.6%) | 3 703 (61.7%) |
| Charlson Index (categories) |  |  |
| 0 | 651 386 (63.4%) | 2 964 (49.4%) |
| 1, 2 | 306 349 (29.8%) | 2 048 (34.1%) |
| 3, 4 | 42 433 (4.1%) | 499 (8.3%) |
| >= 5 | 27 855 (2.7%) | 487 (8.1%) |

**Supplementary Table 17: Broad-spectrum antibiotics**

Beta regression model for the prescription rate of broad-spectrum antibiotics on practices level. Documented are the effect estimate, the corresponding confidence interval (CI) limits, standard errors, and the p-value.

| Covariate | Effect estimate | Lower CI limit | Upper CI limit | St. error | p-value |
| --- | --- | --- | --- | --- | --- |
| Otolaryngologist vs. General Practitioner | -0.045 | -0.125 | 0.035 | 0.041 | 0.269 |
| Pediatrician vs. General Practitioner | -0.668 | -0.741 | -0.595 | 0.037 | <0.001 |
| Gynecologist vs. General Practitioner | -0.175 | -0.231 | -0.120 | 0.028 | <0.001 |
| Urologist vs. General Practitioner | 0.423 | 0.333 | 0.512 | 0.046 | <0.001 |
| Other spec. groups vs. General Practitioner | 0.251 | 0.162 | 0.341 | 0.046 | <0.001 |
| Urbanization vs. Rural location | 0.164 | -0.022 | 0.350 | 0.095 | 0.085 |
| Urban vs. rural location | 0.584 | 0.327 | 0.337 | 0.012 | <0.001 |

**Supplementary Table 18: Primary and secondary outcomes contrasted for gender**

|  | Male | Female |
| --- | --- | --- |
| Acute non-complicated infections |  |  |
| N | 1 484 923 | 1 644 366 |
| N (%) antibiotics | 439 222 (29.6%) | 553 602 (33.7%) |
| Quinolone prescriptions for acute non-complicated infections and antibiotics |  |  |
| N | 458 490 | 575 531 |
| N (%) quinolones | 33 613 (7.3%) | 49 908 (8.7%) |
| Recommended antibiotic prescriptions for acute upper respiratory tract infection and antibiotics |  |  |
| N | 224 118 | 289 038 |
| N (%) recommended antibiotics | 52 769 (23.5%) | 60 340 (20.9%) |
| Recommended antibiotic prescriptions for acute bronchitis and antibiotics |  |  |
| N | 151 273 | 181 823 |
| N (%) recommended antibiotics | 29 438 (19.5%) | 32 189 (17.7%) |
| N (%) alternative antibiotics | 65 697 (43.4%) | 79 931 (44.0%) |
| Recommended antibiotic prescriptions for sinusitis and antibiotics |  |  |
| N | 40 098 | 63 256 |
| N (%) recommended antibiotics | 7 867 (19.6%) | 11 490 (18.2%) |
| N (%) alternative antibiotics | 16 336 (40.7%) | 25 646 (40.5%) |
| Recommended antibiotic prescriptions for tonsillitis and antibiotics |  |  |
| N | 103 838 | 125 043 |
| N (%) recommended antibiotics | 25 115 (24.2%) | 29 826 (23.9%) |
| N (%) alternative antibiotics | 4 203 (4.0%) | 4 565 (3.7%) |
| Recommended antibiotic prescriptions for otitis media and antibiotics |  |  |
| N | 42 830 | 49 438 |
| N (%) recommended antibiotics | 18 280 (42.7%) | 19 495 (39.4%) |
| N (%) alternative antibiotics | 13 930 (32.5%) | 15 969 (32.3%) |

**Supplementary Table 19: Primary and secondary outcomes contrasted for DMP**

|  | Any DMP no | Any DMP yes |
| --- | --- | --- |
| Acute non-complicated infections |  |  |
| N | 582 270 | 2 547 019 |
| N (%) antibiotics | 175 680 (30.2%) | 817 144 (32.1%) |
| Quinolone prescriptions for acute non-complicated infections and antibiotics |  |  |
| N | 184 185 | 849 836 |
| N (%) quinolones | 10 511 (5.7%) | 73 010 (8.6%) |
| Recommended antibiotic prescriptions for acute upper respiratory tract infection and antibiotics |  |  |
| N | 103 981 | 409 175 |
| N (%) recommended antibiotics | 27 137 (26.1%) | 85 972 (21.0%) |
| Recommended antibiotic prescriptions for acute bronchitis and antibiotics |  |  |
| N | 25 438 | 307 658 |
| N (%) recommended antibiotics | 4 818 (18.9%) | 56 809 (18.5%) |
| N (%) alternative antibiotics | 11 668 (45.9%) | 133 960 (43.5%) |
| Recommended antibiotic prescriptions for sinusitis and antibiotics |  |  |
| N | 32 721 | 70 633 |
| N (%) recommended antibiotics | 5 710 (17.5%) | 13 647 (19.3%) |
| N (%) alternative antibiotics | 16 232 (49.6%) | 25 750 (36.5%) |
| Recommended antibiotic prescriptions for tonsillitis and antibiotics |  |  |
| N | 44 365 | 184 516 |
| N (%) recommended antibiotics | 8 263 (18.6%) | 46 678 (25.3%) |
| N (%) alternative antibiotics | 2 230 (5.0%) | 6 538 (3.5%) |
| Recommended antibiotic prescriptions for otitis media and antibiotics |  |  |
| N | 29 587 | 62 681 |
| N (%) recommended antibiotics | 12 192 (41.2%) | 25 583 (40.8%) |
| N (%) alternative antibiotics | 9 944 (33.6%) | 19 955 (31.8%) |

**Supplementary Table 20: Primary and secondary outcomes contrasted for Charlson Index**

|  | Charlson Index | | | |
| --- | --- | --- | --- | --- |
|  | 0 | 1, 2 | 3, 4 | >= 5 |
| Acute non-complicated infections |  |  |  |  |
| N | 2 188 488 | 763 434 | 106 474 | 70 893 |
| N (%) antibiotics | 618 454 (28.3%) | 303 590 (39.8%) | 42 630 (40.0%) | 28 150 (39.7%) |
| Quinolone prescriptions for acute non-complicated infections and antibiotics |  |  |  |  |
| N | 654 350 | 308 397 | 42 932 | 28 342 |
| N (%) quinolones | 38 227 (5.8%) | 33 685 (10.9%) | 6 703 (15.6%) | 4 906 (17.3%) |
| Recommended antibiotic prescriptions for acute upper respiratory tract infection and antibiotics |  |  |  |  |
| N | 343 602 | 134 134 | 20 836 | 14 584 |
| N (%) recommended antibiotics | 81 035 (23.6%) | 26 330 (19.6%) | 3 493 (16.8%) | 2 251 (15.4%) |
| Recommended antibiotic prescriptions for acute bronchitis and antibiotics |  |  |  |  |
| N | 121 539 | 176 769 | 21 863 | 12 925 |
| N (%) recommended antibiotics | 23 690 (19.5%) | 32 099 (18.2%) | 3 790 (17.3%) | 2 048 (15.8%) |
| N (%) alternative antibiotics | 55 141 (45.4%) | 77 674 (43.9%) | 8 350 (38.2%) | 4 463 (34.5%) |
| Recommended antibiotic prescriptions for sinusitis and antibiotics |  |  |  |  |
| N | 69 935 | 26 729 | 4 107 | 2 583 |
| N (%) recommended antibiotics | 13 451 (19.2%) | 4 778 (17.9%) | 701 (17.1%) | 427 (16.5%) |
| N (%) alternative antibiotics | 27 894 (39.9%) | 11 113 (41.6%) | 1 808 (44.0%) | 1 167 (45.2%) |
| Recommended antibiotic prescriptions for tonsillitis and antibiotics |  |  |  |  |
| N | 189 657 | 34 818 | 2 691 | 1 715 |
| N (%) recommended antibiotics | 47 960 (25.3%) | 6 335 (18.2%) | 412 (15.3%) | 234 (13.6%) |
| N (%) alternative antibiotics | 7 601 (4.0%) | 1 098 (3.2%) | 48 (1.8%) | 21 (1.2%) |
| Recommended antibiotic prescriptions for otitis media and antibiotics |  |  |  |  |
| N | 72 744 | 16 388 | 1 862 | 1 274 |
| N (%) recommended antibiotics | 31 603 (43.4%) | 5 483 (33.5%) | 424 (22.8%) | 265 (20.8%) |
| N (%) alternative antibiotics | 23 293 (32.0%) | 5 495 (33.5%) | 636 (34.2%) | 475 (37.3%) |
